# Do sex and gender matter in the pharmacological treatment of depression?

**DOI:** 10.1007/s00210-025-04611-z

**Published:** 2025-10-23

**Authors:** Johanna Seifert

**Affiliations:** Department of Psychiatry, Social Psychiatry and Psychotherapy, Hannover Medical School, Carl-Neuberg-Straße 1, 30625 Hannover, Germany

**Keywords:** Depressive disorders, Antidepressant agents, Sex factors, Estrogens, Testosterone, Drug-related side effects and adverse reactions

## Abstract

Depression shows significant differences in prevalence, symptomology and treatment outcomes in men and women. Women have a twofold higher risk of depression than men and often present with different depression subtypes. These differences are at least partly mediated by gender roles as well as biological differences, especially regarding the effects sex hormones, which may also impact the efficacy and tolerability of antidepressant drugs (ADDs). This narrative review examines how both biological sex and gender affect the occurrence and presentation of depression as well as the pharmacodynamics and pharmacokinetics of ADDs, focusing on hormonal modulation, treatment efficacy, adverse drug reactions (ADRs), placebo response and sex-/gender-specific symptom presentation of depression. Relevant research pertaining to the objective of this narrative review was extracted in a non-systematic manner. Although many interactions between sex hormones and serotonergic and noradrengic neurotransmission have been described, they rarely translate into robust clinical implications that allow for sex-specific treatment recommendations. The neurosteroid allopregnanolone, however, has been exclusively approved for postpartum depression. Sex hormones, especially estrogen, also impact drug metabolism, which is relevant particularly during pregnancy. Regarding ADRs, women are less willing to tolerate weight gain, whereas men suffer more from sexual dysfunction. Whether or not placebo- and nocebo-response to ADDs is subject to sex/gender differences is currently unknown. Current research does not allow any conclusive recommendations in the treatment of depression according to sex and gender. Future research should prioritize sex-stratified analyses to better comprehend and address these biological differences.

## Introduction

Depression is a debilitating mental disorder presenting with the two cardinal symptoms persistent low mood and loss of interest or pleasure, accompanied by a range of additional symptoms including insomnia, loss of appetite, feelings of worthlessness and suicidal ideation (American Psychiatric Association 2013). Over the past three decades, the global incidence of depression has increased by 50% (Liu et al. 2020) and is currently the second leading cause of disability worldwide (Global Burden of Disease 2021). Prevalence rates vary by region, with lifetime and 12-month prevalence in high-income countries estimated at 14.6% and 5.5%, respectively, compared to 11.1% and 5.9% in low- and middle-income countries (Lim et al. 2018). From an economic perspective, depression also imposes a substantial burden, due to both direct treatment costs and indirect costs for example due to loss of productivity (König et al. 2019).

Epidemiological data suggest that sex/gender differences in depression begin to emerge during adolescence. While boys under the age of 13 appear slightly more likely to report symptoms of depression than girls, the consistently higher burden of depression in females emerges with the onset of puberty (Twenge and Nolen-Hoeksema 2002). In adulthood, women are twice as likely to be affected by depression as men (Li et al. 2023). Notably, second only to low back pain, depression is the disease with the greatest difference in disability-adjusted life-years between women and men (Patwardhan et al. 2024). Women are also more likely to experience an earlier onset, recurrent episodes and a chronic course of illness (Frackiewicz et al. 2000). Various biological, psychological and social explanations have been proposed to account for this disparity (Kuehner 2017, 2003; Hyde and Mezulis 2020).

Perhaps under-recognized is the fact that sex hormones play an active role in the central nervous system (CNS). Differences in hormone levels, especially of estrogen and progesterone, are thought to contribute to changes in mood, emotion and cognition and may explain why certain mood disorders are more prevalent in women, particularly during hormonally sensitive life stages. The effects of hormonal fluctuations first become apparent during puberty, at which point the sex difference of depression emerges (Hyde and Mezulis 2020). Early puberty has been found to increase the risk of depression in adolescent girls, although with an overall small effect size (Ullsperger and Nikolas 2017). During pregnancy, levels of both estrogen and progesterone substantially increase. Following childbirth, the concentrations of both hormones drop sharply, which – combined with the physical and emotional demands of caring for a newborn – may contribute to the onset of depression (Payne and Maguire 2019). A recent meta-analysis suggests that the prevalence of postpartum depression in otherwise healthy mothers (i. e., without a prior history of depression) is approximately 17% (Shorey et al. 2018). Menopause is another critical life stage driven by hormonal changes, characterized by initial fluctuations followed by a steady decline of estrogen. Women in the perimenopausal and early postmenopausal phases are 2–5 more likely to experience depressive symptoms compared to premenopausal women (Bromberger and Epperson 2018). Similarly, decreasing testosterone concentrations with age in men are associated with a higher prevalence of depression (Almeida et al. 2008).

Apart from hormonal fluctuations, women are often faced with distinct social stressors of the female gender role, including the societal expectations surrounding caregiving, balancing work and family responsibilities, while experiencing lower economic stability, lower educational status and reduced autonomy in comparison to men. They are also more likely to be subjected to physical and sexual violence, both in childhood and adulthood (Kuehner 2003). Further, the ways in which women tend to cope with stress may contribute to the development of depression. Statistically, women are more likely to engage in a ruminative coping style, which involves repetitively and unproductively focusing on negative emotions and stressors. The effects of the gender-specific differences in rumination on subsequent depression appear to be small, a more significant difference is found in co-rumination (i. e., ruminating with another person), which females are more likely to engage in compared to males (Johnson and Whisman 2013; Hyde and Mezulis 2020).

On the other hand, the question has been raised, whether depression is indeed more prevalent in women or if it is simply under-diagnosed in men (Shi et al. 2021). Although females are evidently more likely to report symptoms of depression (Shi et al. 2021), several considerations challenge this observation. Firstly, depression is the leading cause of suicide (Favril et al. 2022) and while women are more likely to attempt suicide, the rate of completed suicide is significantly higher in men (Bommersbach et al. 2022). Sociocultural expectations imposed on men may discourage them from reporting symptoms of depression, such as crying, as these may be perceived as inconsistent with traditional male gender roles. It has also been suggested that current diagnostic criteria for depression are better suited to detect depression in women, who may be more willing to openly present affective symptoms (Shi et al. 2021). Men, on the other hand, may tend to externalize symptoms, resulting in a non-traditional presentation of symptoms including aggressive behavior, irritability, substance abuse, risk taking and reduced impulse control (Möller-Leimkühler et al. 2004; Winkler et al. 2005). Unlike depression, substance use disorders such as of alcohol are consistently more prevalent in men (Glantz et al. 2020), potentially accounting, at least in part, for this disparity.

It is important to bear in mind, that though the terms “sex” and “gender” are related, they are distinct. Sex refers to biological and physiological characteristics that distinguish humans as either male, female or intersex. The basis to determining sex include chromosomes (e. g., XX, XY), reproductive anatomy and sex hormones (e. g., estrogen, testosterone). By contrast, gender refers socially constructed identities, roles, behaviors and expectations. While in a majority of people, sex and gender overlap (e. g., person with female sex also identifies as a women), this is not always the case, adding a layer of complexity when considering sex and gender aspects (Christiansen et al. 2022). In some regards it is fairly easy to distinguish sex and gender, for example, when considering the effects of sex hormones. The interplay of sex and gender becomes more entangled, such as when discussing adverse effects. Are women more prone to antidepressant-induced weight gain due to their biological profile, are they more bothered by it as a result of body image mediated by gender expectations or is it an interplay of both? In the following article, both sex and gender will be discussed, as both are relevant. In order to provide a precise nomenclature, the term “sex” will be used when referring exclusively to biological factors, “gender” when referring exclusively to sociocultural concepts and “sex/gender” when the two entities cannot be clearly separated.

Overall, it appears that sex, gender and depression interact in a complex manner with regard to prevalence, trajectory and symptom presentation. These observations suggest that sex- and gender-specific considerations may also play a significant role in the pharmacological treatment of depression, especially regarding treatment response. The following narrative review will explore different aspects of antidepressant drug (ADD) response and presentation of depression based on sex and gender. As it provides a basis for further discussion, the different mechanisms of action of ADDs will briefly be presented (Part 1). Then, biologically determined differences in neurotransmission mediated by sex hormones, their potential impact on antidepressant therapy and clinical implications will be explored, before further considering sex-specific differences in ADD pharmacokinetics (Part 2). After exploring these largely biologically determined aspects, the concepts of sex and gender will become more difficult to separate when analyzing other clinically relevant aspects that effect ADD treatment, including adverse drug reactions (ADRs) and placebo- and nocebo-response (Part 3). However, due to the great complexity of depression and antidepressant response, the present manuscript cannot comprehensively assess all aspects that may contribute to sex- and gender-specific differences.

## Part 1: Mechanisms of action of antidepressant drugs

**Fig. 1:**
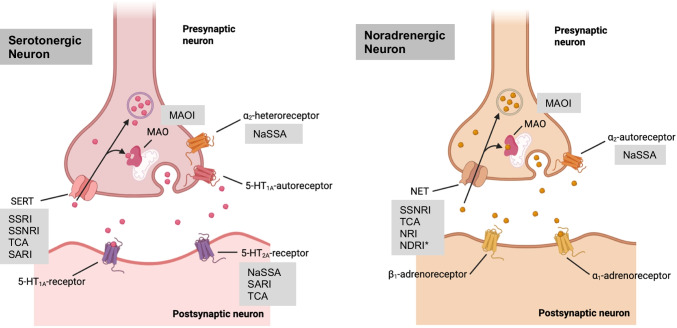
Schematic image depicting a serotonergic and noradrenergic neuron with relevant targets of antidepressant drugs. Image created using biorender.com. SERT, serotonin transporter; NET, norepinephrine transporter; SSRI, selective serotonin reuptake inhibitor; SSNRI, selective serotonin-norepinephrine reuptake inhibitor; TCA, tricyclic antidepressant; MAO, monoamine oxidase; MAOI, monoamine oxidase inhibitor; NaSSA, noradrenergic and specific serotonergic antidepressant; NDRI, norepinephrine-dopamine reuptake inhibitor; SARI, serotonin antagonist and reuptake inhibitor. *NDRI further block the dopamine transporter (DAT)

When faced with the challenge of treating depression, clinicians and patients can choose from an array of more than 20 different drugs approved for this indication. The majority of ADDs enhance neurotransmission of serotonin and/or norepinephrine. One of the only exceptions is currently esketamine, the first “rapid-acting” antidepressant, which acts on the glutamate system (LeGates et al. 2019). Figure 1 provides an overview of the different classes of antidepressant drugs and their therapeutic targets.

### Inhibition of the reuptake of serotonin, norepinephrine and dopamine

A common mechanism of ADDs is the inhibition of monoamine transporters located on the presynaptic membrane. Specific transporters remove serotonin (via the serotonin transporter, SERT), norepinephrine (via the norepinephrine transporter, NET) and dopamine (via the dopamine transporter, DAT) from the synaptic cleft. This process terminates the action of monoamines on pre- and postsynaptic receptors and allows them to be recycled or broken down. Blocking these transporters increases the availability of monoamines in the synaptic cleft. This mechanism is utilized by several classes of ADDs, i. e., selective serotonin reuptake inhibitors (SSRI; inhibition of SERT), selective serotonin-norepinephrine reuptake inhibitors (SSNRI; inhibition of SERT and NET), norepinephrine reuptake inhibitors (NRI; inhibition of NET), norepinephrine-dopamine reuptake inhibitors (NDRI, e. g., bupropion; inhibition of NET and DAT) and tricyclic antidepressants (TCA; inhibition of SERT and NET) (Stahl 2021).

### Inhibition of monoamine oxidase

Monoamine oxidase inhibitors (MAOI) also increase the availability of monoamines including serotonin, norepinephrine and dopamine. They achieve this by inhibiting monoamine oxidase (MAO), an enzyme located on the outer mitochondrial membrane within neurons that is responsible for degrading and therefore inactivating monoamines. By inhibiting this enzyme, MAOI prevent the breakdown of monoamines, resulting in increased concentrations in the brain. While tranylcypromine acts as an irreversible inhibitor of MAO type A and B, moclobemide is a reversible inhibitor of MAO type B (Stahl 2021).

### Blocking of serotonin receptors

Of the known 15 subtypes of serotonin (5-HT) receptors, the 1 A and 2 A subtypes appear to be particularly significant in the pathophysiology of depression. 5-HT_1A_ receptors located on the presynaptic membrane serve as autoreceptors. When activated, they inhibit the release of serotonin, serving as a feedback mechanism to regulate serotonin levels within the synaptic cleft. Although there currently no drugs that specifically block this receptor subtype, the administration of SSRI leads to a rapid desensitization of 5-HT_1A_ autoreceptors, indirectly enhancing serotonergic neurotransmission. Activation of postsynaptic 5-HT_2A_ receptors, on the other hand, is associated with negative effects on mood (Fakhoury 2016). Consequently, drugs with antagonistic effects on this receptor, such as mirtazapine, a noradrenergic and specific serotonergic antidepressant (NaSSA), and trazodone, a serotonin antagonist and reuptake inhibitor (SARI), are associated with antidepressant effects (Fakhoury 2016; Stahl 2009).

### Blocking of adrenoreceptors

Similar to serotonin receptors, different types of adrenoreceptors are located on both the presynaptic and postsynaptic membranes. The presynaptic α_2_-adrenoreceptor normally functions to inhibit the release of norepinephrine and serotonin into the synaptic cleft. Drugs that block this receptor, such as mirtazapine, therefore increase the release of both neurotransmitters, thereby producing antidepressant effects (Stahl 2009). No drugs currently target postsynaptic adrenoreceptors specifically for the treatment of depression.

### Activation of melatonin receptors

In a more recent development in psychopharmacology, agomelatine has been approved for the treatment of depression. Agomelatine acts as an agonist at the melatonergic receptor subtypes MT_1_ and MT_2_ and as an antagonist at 5-HT_2C_ receptors. It exerts antidepressant effects and is meant to restore the circadian rhythm (Racagni et al. 2011).

### Antagonism at glutamatergic receptors

The only ADD that exerts its effects through glutamatergic neurotransmission is the rapid-acting ADD (es)ketamine, which is an antagonist at glutamatergic N-methyl-D-aspartate (NMDA) receptors. However, the antidepressant effects of (es)ketamine are complex and may not be primarily mediated by this mechanism alone. By blocking the NDMA receptor, a surge in glutamate transmission occurs, which in turn stimulates the glutamatergic α-amino-3-hydroxy-5-methyl-4-isoxazolepropionic acid (AMPA) receptor. This stimulation appears to promote the release of brain derived neurotropic factor (BDNF), a protein associated with enhanced synaptic plasticity and increased synthesis of synaptic proteins, thereby contributing to the restoration of synaptic function (Duman et al. 2016). Unlike all other traditional ADDs that effect serotonergic and noradrengic neurotransmission, the antidepressant effects of (es)ketamine can manifest within hours of application (Molero et al. 2018).

### Antidepressant drug-induced neuroplasticity

It is not the seemingly simple increase in the availability of serotonin, norepinephrine or dopamine that directly accounts for the antidepressants effects of ADDs. If that were the case, the therapeutic effects of ADDs such as SSRIs and SSNRIs would manifest within hours of administration rather than requiring several weeks of continuous use. By enhancing serotonergic and/or noradrenergic neurotransmission, ADDs subsequently induce a cascade of changes, including the downregulation and desensitization of certain receptor postsynaptic subtypes (e. g., 5-HT_1A_, 5-HT_2A_, β-adrenergic receptors) and an upregulation of 5-HT_1A_ autoreceptors (Celada et al. 2004; Zhang et al. 2022). Moreover, long-term treatment increases the expression of BDNF, promoting synaptic plasticity and neurogenesis (Martinowich and Lu 2008). The stimulation of BDNF release by traditional ADDs is a gradual process which takes weeks, unlike the BDNF release-enhancing effects of (es)ketamine, which have a more direct and rapid onset (Duman et al. 2016).

## Part 2: Biologically determined sex-specific differences in neurobiology and drug metabolism of antidepressant drugs

### Sex hormones in depression and antidepressant response: neurobiological and clinical implications

One of the fundamental differences between males and females is the presence and availability of different sex hormones. Although estrogen and progesterone are considered “female sex hormones” and androgen and testosterone are the “male sex hormones”, all of them are found in both men and women and have a common synthesis pathway originating from cholesterol (Hansen et al. 2017). Apart from their well-known roles in regulating sexual development, reproduction and secondary sexual characteristics, sex hormones play an active role in the CNS and regulate mood, cognition, behavior, pain, stress response and neuroprotection. They are mainly produced by the endocrine glands in the ovaries and the testes and can cross the blood–brain barrier due to their lipophilic nature. In the brain, sex hormones can bind to specific sex hormone receptors that are scattered throughout various regions of the brain. The mechanisms of sex hormone receptor activation in the CNS are complex; an overview on this topic can be found elsewhere (McEwen and Milner 2017). One of the main ways that steroid hormones work in the CNS is via binding to classical intracellular receptors that in the nucleus act as transcription factors which regulate gene expression. This type of receptor is found particularly in the hypothalamus and hippocampus in neurons and glial cells. Examples for this type of receptor are estrogen receptors (ERα, ERβ), androgen receptors and progesterone receptors. Binding to these receptors results in long-term effects, such as modulation of neurotransmitter receptor expression. In so, the sex hormone signaling pathways in the CNS activate and interact with different neurotransmitter systems, such as serotonin, norepinephrine and glutamate (McEwen and Milner 2017; Del Río et al. 2018).

In addition to transport from the periphery, some sex hormones and their active metabolites can be synthesized de novo in the brain from cholesterol or other steroid precursors. The most well-known neurosteroid is a substance called allopregnanolone, which is derived from progesterone (Schumacher et al. 2014). Unlike the classical steroid hormones from the periphery which bind to specific hormone receptors, neurosteroids exert their effects by directly modulating neurotransmitter receptors, mainly GABA (e. g., allopregnanlone) and NMDA (e. g., dihydroepiandrosterone [DHEA]). These non-genomic mechanisms quickly exert effects on different brain functions (Maguire and Mennerick 2024; Nenezic et al. 2023).

The complex actions of endogenous sex hormones in the CNS, their effects on the development of depressive symptoms and their potential interactions with ADDs will be discussed in the following. Previous research describes an abundance of interactions between sex steroids produced by the adrenal glands (e. g., estrogen, progesterone, androgen, testosterone), neurosteroids (e. g., allopregnanolone, DHEA) and various targets within the serotonergic and noradrenergic systems. However, most of these effects currently aren’t associated with clinically impactful implications for treatment. Even when benefits are observed, they appear somewhat speculative only supported by conflicting evidence and are generally limited to specific and narrowly defined clinical contexts. The most significant “sex-specific” treatment that is of sound clinical significance entails the use of allopregnanolone in postpartum depression, a condition which in itself is uniquely female.

#### Estrogen

Estrogen, mainly in the form of estradiol, has multiple effects on the serotonin system. Firstly, it upregulates tryptophan hydroxylase, the enzyme responsible for the synthesis of serotonin. Greater availability of this enzyme results in a higher production of serotonin (Bendis et al. 2024). Further, it has been observed that estrogen upregulates the SERT. While this may seem counterintuitive in the context of SSRIs considering these drugs prevent the SERT from removing serotonin from the synaptic cleft, this upregulation has been associated with a boost in serotonin turnover and synthesis, especially after an extended period of low estrogen, such as post-menopause or post-partum (Hernández-Hernández et al. 2019). Studies have shown that not only does SERT density increase after prolonged exposure to SSRIs, a higher baseline density of SERTs in certain regions of the brain may contribute to higher residual binding of SSRIs (Baldinger et al. 2014). Further, a higher SERT occupancy and baseline binding has been associated with clinical response to treatment with SSRI (Lanzenberger et al. 2012). Finally, estrogen modulates the activity of presynaptic 5-HT_1A_ (downregulation) and postsynaptic 5-HT_2A_ receptors (upregulation). An increased level of 5-HT_2A_ receptors may induce a desensitization of presynaptic 5-HT_1A_ autoreceptors, which are in turn unable to reduce serotonin synthesis. As a result, serotonin concentrations in the synaptic cleft are increased (Rybaczyk et al. 2005).

These complementary serotonergic mechanisms may explain why some studies have found a superior response to ADDs that selectively target the serotonergic system, such as SSRIs, in females. The supposed superior efficacy of SSRIs in females is one of the most consistently described findings in regards to sex-specific treatment approaches in depression (Kornstein et al. 2000; Khan et al. 2005; Berlanga and Flores-Ramos 2006; Young et al. 2009), although there is an at least equally extensive body of conflicting evidence (Quitkin et al. 2002; Hildebrandt et al. 2003; Parker et al. 2003; Thiels et al. 2005). An older review featuring a meta-analytical approach including eight randomized controlled trials (RCTs; *N* = 2045) from 2001 concluded that men and women of all age groups respond comparably to both SSRIs and venlafaxine (Entsuah et al. 2001). However, underscoring the potential relevance of estrogen in the response to serotonergic ADDs, a recent meta-analysis on the treatment of depression in peri- and post-menopausal women aggregated 75 RCTs (*N* = 19,048). The authors concluded that the therapeutic use of hormone replacement therapy, including oral and transdermal estrogen, showed significant antidepressant effects both in monotherapy, as well as in combination with an SSRI (specifically fluoxetine, though evidence stems from only two studies). These superior effects were limited to peri-menopausal women with a definite diagnosis of depression, whereas post-menopausal women responded less consistently to hormone replacement therapy (Tseng et al. 2023).

It appears that estrogen not only interacts with serotonin, but also with noradrenergic neurotransmission by increasing the synthesis of norepinephrine and downregulating its degradation. Estrogen also alters the density of adrenergic receptors, particularly postsynaptic β-adrenergic receptors, limiting overstimulation with norepinephrine and balancing higher norepinephrine concentrations with receptor downregulation. These effects appear to fluctuate during the female cycle and are particularly prominent during high-estrogen phases (Eker et al. 2009). These effects of estrogen on noradrenergic neurotransmission suggest that it may work synergistically with the mechanisms of ADDs with noradrenergic properties. However, these theoretical implications fail to translate in clinical practice, as sex-specific difference in the response to norepinephrine-based ADDs are not consistently observed in clinical studies (Khan et al. 2005; Kornstein et al. 2010, 2014). Berlanga and Flores-Ramos previously aimed to assess this matter by comparing the sex-specific drug response to citalopram and reboxetine (Berlanga and Flores-Ramos 2006), a norepinephrine reuptake inhibitor that selectively binds to the NET (Hajós et al. 2004). The authors found a superior treatment response of pre-menopausal women to the SSRI citalopram, however, reboxetine did not show a favorable response in either sex (Berlanga and Flores-Ramos 2006).

#### Progesterone

As with estrogen, progesterone also modulates neurotransmission relevant to depression, although in an unfavorable way. High levels of progesterone, such as during the luteal phase of the menstrual cycle, have been associated with mood instability and anxiety. This is thought to be due to the primarily suppressive effects of progesterone on the serotonin system (Freeman 2015; McEwen and Milner 2017), working in tandem with estradiol to maintain homeostasis. While estradiol, for example, promotes synaptogenesis, progesterone terminates or reverses these effects (Woolley and McEwen 1993). Because of progesterone’s adverse impact on mood, its use was considered contraindicated in patients with affective disorders. This, however, changed with the establishment of the role of the naturally occurring progesterone metabolite and neurosteroid allopregnanolone. Allopregnanolone, perhaps better known as brexanolone, was approved by the United State Food and Drug Administration (FDA) in 2019 for the treatment of postpartum depression. The concentration of endogenous brexanolone rapidly decreases following childbirth. In order to restore allopregnanolone signaling, synthetic brexanolone is administered intravenously over the course of 60 h with continuous monitoring, relieving symptoms of post-partum depression within hours by enhancing the activity of γ-aminobutyric acid (GABA) type A receptors (Reddy et al. 2023). A more recent development in the treatment of postpartum depression is zuranolone, a structurally modified synthetic analog of allopreganolone. As of 2023, the FDA approved zuralonone for treatment of postpartum depression. Unlike brexanolone, zuranlone can be administered orally over the course of two weeks without hospital-based monitoring, improving its accessibility and lowering treatment costs (Nashwan et al. 2024).

#### Androgen and testosterone

The two-fold higher prevalence of major depressive disorder in women suggests that physiological concentrations of male sex hormones may be protective. Indeed, androgens are associated with antidepressant and neuroprotective actions in several brain regions that regulate mood (Hauger et al. 2022). Androgens can induce the upregulation of SERT expression and increase the firing rate of serotonergic neurons, both resulting in a greater serotonergic neurotransmission, which are associated with antidepressant effects (Nautiyal and Hen 2017). The paradoxical effects of higher SERT density and SSRI response have been outlined above (see section on estrogen). Supporting this is research that shows that low levels of testosterone – such as in patients with hypogonadism, during treatment with androgen receptor antagonists (e. g., as treatment for prostate cancer) or as men age – significantly correlate with depressive symptoms in men. There are also studies demonstrating a beneficial response of testosterone replacement therapy (TRT) in hypogonadal men with depression (Hauger et al. 2022). A recent systematic review on the effects of TRT on depressive symptoms in men with late-onset testosterone deficiency included a total of 15 RCTs (*N* = 1586) and deduced that a positive response to TRT was limited to those with pretreatment depression of mild severity, but not in men with clinically relevant major depressive disorders. In men without prior depression, TRT was shown to reduce symptom scores, although clinical relevance of this effect remained uncertain (Vartolomei et al. 2020). In so, evidence supporting the use of TRT to treat clinically relevant depression in males – even in the presence of testosterone deficiency – is not convincing (Bhasin and Seidman 2019).

DHEA, a precursor in the biosynthesis of androgens and testosterone, and its sulfate ester DHEA-S act as neurosteroids in the CNS. While allopregnanolone has very pronounced effects on GABA-A receptors, DHEA demonstrates only a modest negative modulation of this receptor subtype. Instead, DHEA binds to central sigma-1 receptors, which indirectly positively modulate NMDA receptors and potentiate glutamatergic neurotransmission (Nenezic et al. 2023). Further, by increasing the concentration of BDNF and expression of 5-HT_2A_ receptors in the amygdala, DHEA has the potential to induce anxiolytic and antidepressant effects (Sripada et al. 2014). Supporting these positive effects of DHEA, studies have shown that lower levels of DHEA are associated with depression (Souza-Teodoro et al. 2022). Because of these beneficial effects, exogenous DHEA has been studied as a therapeutic agent for the treatment of depression in both men and women. A meta-analysis examining the efficacy of DHEA in the treatment of depression was only able to include two studies (*N* = 48). Both of the studies yielded positive results in improving depression, with only minimal side effects (Peixoto et al. 2018). A recent study from 2024 suggests that higher concentrations of DHEA-S are associated with better treatment outcomes with ADDs (Souza-Teodoro et al. 2024). However, research in this area is largely lacking, especially in comparison to the very well-established efficacy of allopregnanlone replacement therapy in postpartum depression. Whether DHEA supplementation is beneficial as a monotherapy as an alternative to traditional ADDs, or whether it has the capacity to modulate the efficacy of ADDs, remains poorly studied.

#### Putative effects of synthetic sex hormones

The observation that sex hormones complexly affect the CNS makes it plausible to assume that the introduction of synthetic hormones in the form of hormonal contraceptives and gender-affirming hormone treatment may also do so. Hormonal contraceptives may improve mood in some women but worsen it in others (Pletzer et al. 2023; Mu and Kulkarni 2022). However, research on the effect of hormonal contraceptives on ADDs and their efficacy is currently severely lacking. In transgender individuals, among whom the prevalence of depression and anxiety is significantly higher than in cisgender individuals, gender-affirming hormone treatment generally improves mental health. However, it may also mediate the risk of depression or interact with ADDs, although these drug-hormone interactions currently remain poorly characterized (Kalayjian et al. 2024).

#### Sex hormones and non-serotonergic/noradrengic antidepressants

Agomelatine primarily acts as a melatonin receptor agonist (Racagni et al. 2011). Some data indicates sex differences in the circadian profiles of melatonin: Estrogen and progesterone may suppress melatonin, whereas testosterone may help maintain nocturnal peaks (Cipolla-Neto et al. 2022). Although rodent studies suggest superior anxiolytic effects in male rats (Regenass et al. 2018), sex-related differences in efficacy in humas remain speculative. For the rapid-acting, non-serotonergic ADD esketamine, a majority of studies report no significant sex differences in treatment response (Medeiros et al. 2024).

### Sex-dependent pharmacokinetics of antidepressant drugs

Apart from modulating neurotransmission, sex hormones can also influence the pharmacokinetic properties of drugs, i. e., absorption, distribution, metabolism and excretion (overview in Damoiseaux et al. 2014 and Kokras et al. 2011), and in so the efficacy and tolerability of drugs. As briefly mentioned previously, several studies have indicated a superior effect of TCAs in men in comparison to women (Kornstein et al. 2000; Hamilton 1996; Frank et al. 1988; Raskin 1974). While it is still not fully clear whether or not men respond preferentially to TCAs, studies that detected a difference in TCA response mainly argued it was due to differences in drug metabolism. Some evidence has pointed out higher plasma levels of TCAs in women (Gex-Fabry et al. 1990; Hildebrandt et al. 2003; Dahl et al. 1996; Unterecker et al. 2013; Billups et al. 2009), even when correcting for dose (Unterecker et al. 2013). However, these effects are not limited to TCAs but have also been described for many other ADDs, including venlafaxine, citalopram, sertraline and mirtazapine (Unterecker et al. 2012). These factors may especially reduce tolerability, overall willingness to take a drug and, of course, a patient’s response to treatment. In the following, relevant parameters of the pharmacokinetics of ADDs are presented and discussed. Of note, many of the sex-dependent observations remain of theoretical nature and of unknown significance for the metabolism of ADDs. As a result, general recommendations on ADD treatment based on sex differences in pharmacokinetics are currently not possible (Kokras et al. 2011; Damoiseaux et al. 2014).

#### Absorption

Women have a higher stomach pH, which in turn may increase the bioavailability of ADDs, most of which are weak bases (Damoiseaux et al. 2014). This effect may be additionally reinforced by a slower gut transit time in females, especially in the luteal phase of the menstrual cycle when progesterone levels are high (Coquoz et al. 2022). Whether or not these effects are of clinical significance remains controversial (Kokras et al. 2011; Damoiseaux et al. 2014).

#### Distribution

In order to cross the blood–brain-barrier, ADDs need to be lipophilic (Zheng et al. 2016). Due to the generally higher body fat percentage in women, females may have a greater volume of distribution for ADDs (Damoiseaux et al. 2014). This, in theory, bears potential for increased drug storage and slower release. Moreover, estrogen influences protein binding of ADDs, which may result in changes in drug availability depending on the menstrual cycle phase (Kokras et al. 2011).

#### Metabolism

The sex-dependent variability in metabolism via cytochrome P450 (CYP) enzymes, which most ADDs rely on for their metabolism, is thought to be one of the main reasons for sex-specific differences in pharmacokinetics (Kokras et al. 2011). Several studies have demonstrated a higher activity of the CYP isoenzyme 3A4 in women (Kokras et al. 2011) as a result of an estradiol-mediated upregulation. This effect also applies to CYP2A6 and 2B6, whereas CYP1A2 (Jeong 2010) and 2C19 are down-regulated (Mwinyi et al. 2010). Therefore, hormone fluctuations, for example during the menstrual cycle or during pregnancy, can potentially affect the activity of CYP enzymes and the metabolism of ADDs. It remains unclear, whether the comparatively rapid hormone fluctuations during the course of a menstrual cycle result in clinically significant alterations in drug metabolism (Kashuba and Nafziger 1998). However, the sex hormone-dependent changes in drug metabolism are a relevant consideration during pregnancy and have found a considerable amount of attention (overview in Paulzen and Schoretsanitis 2023; Schoretsanitis et al. 2020), especially since 1 in 12 women use an ADD at some point during their pregnancy (Huybrechts et al. 2013). As a result, the dose of (antidepressant) drugs metabolized by the effected CYP enzymes require adjusting and, perhaps counterintuitively, higher doses may be needed in order to reach therapeutic drug concentrations in pregnant women. Accordingly, researchers recommend the implementation of therapeutic drug monitoring under this circumstance (Paulzen and Schoretsanitis 2023; Schoretsanitis et al. 2020).

#### Elimination

Excretion of drugs occurs either via renal or hepatic elimination and therefore mainly depends on the rate of blood flow into the liver and kidney. Although organ size, blood flow rates and glomerular filtration rates are consistently lower in women, it remains unclear, whether these effects effect the elimination of ADDs in a clinically relevant manner (Kokras et al. 2011; Damoiseaux et al. 2014).

#### Effect of synthetic hormones on the pharmacokinetics of antidepressant drugs

Although frequently concomitantly used, research on the interactions between synthetic hormones – e. g., hormonal contraceptives or gender-affirming hormone treatment – and ADDs and the resulting safety and efficacy concerns for either drug class unfortunately appears somewhat lacking (Berry-Bibee et al. 2016; Schoretsanitis et al. 2022; Cirrincione and Huang 2021). Most hormonal contraceptives are primarily metabolized via CYP3A4 (Schoretsanitis et al. 2022), while most of the commonly used ADDs are metabolized via CYP2D6 and CYP2C19 (Hiemke et al. 2018). Overall, the available evidence suggests low concern for clinically significant interactions between ADDs and hormonal contraceptives (Berry-Bibee et al. 2016). In patients receiving gender-affirming hormone treatment, the synthetic hormones (e. g., testosterone, estrogens) have the same effects on inhibition and induction of CYP enzymes as the endogenous hormones do. This means that potential pharmacokinetic drug-hormone interactions are primarily a concern for patients treated with estrogens, which have a certain propensity to induce or inhibit different CYP enzymes as explained above (Cirrincione and Huang 2021).

## Part 3: Tolerability and response to antidepressant drugs according to sex and gender

### Sex and gender differences in adverse drug reactions

Without a doubt, the consideration of potential ADRs is perhaps one of the most fundamental aspects of deciding which drug best suits a patient. ADRs are one of the most common reasons for patient non-adherence to psychotropic drugs (Semahegn et al. 2020). Women are not only more likely to report ADRs, the spectrum of ADRs also shows a sex disparity (Brabete et al. 2022; Lacroix et al. 2023) and the willingness to tolerate certain ADR may also differ between men and women. Two ADRs of particular interest in this regard are weight gain and sexual dysfunction (Haack et al. 2009; Zimmerman et al. 2005) and their tolerability profile may also largely be mediated by gender roles and norms.

Drug-related weight gain is a legitimate concern for nearly all psychotropic drugs, with the exception of a few drugs, such as bupropion (Gill et al. 2020) and stimulants (Hasnain and Vieweg 2013). Previous research has established several sex-specific differences in brain structure and behavior that influence body weight, suggesting that women have a generally higher risk of becoming obese (Horstmann et al. 2011; Koceva et al. 2024). Whether or not women are also at greater risk of psychotropic drug-induced weight gain remains unclear (Eder et al. 2023; Alonso-Pedrero et al. 2019; Schneider et al. 2020; Gates et al. 2016), it may, however, cause them more discomfort and lead them to report it more often (Seeman 2020; Gates et al. 2016). Dissatisfaction with their body image and composition is common among both women and men. However, any type of weight gain may more significantly interfere with the female tendency to strive more towards a thin body type as often portrayed in media (Want 2009; Koceva et al. 2024). Therefore, preferentially prescribing women ADDs with a lower risk for weight gain may be an advantage in terms of patient satisfaction, treatment adherence and willingness to continue treatment for an extended period of time. One of the most high-risk ADDs for weight gain is mirtazapine (Gill et al. 2020; Schneider et al. 2020) and it can, in fact, be observed that mirtazapine is used significantly less often in the treatment of females both in the psychiatric inpatient (Seifert et al. 2021) and outpatient setting (Sundell et al. 2011). It has been suggested, that the tendency of SSRIs, SSNRIs and TCAs to cause weight gain varies from drug to drug. While amitriptyline, nortriptyline, citalopram and paroxetine appear to have a high risk for this ADR, sertraline, escitalopram and duloxetine are associated with only a moderate risk and bupropion, imipramine, fluoxetine, and venlafaxine have the lowest risk for weight gain (Gill et al. 2020). Next to differences in pharmacokinetics (see section above), the generally higher risk of weight gain for TCAs versus SSRIs and SSNRIs may also contribute to the observation that men may benefit more from treatment with TCAs (Kornstein et al. 2000; Hamilton 1996; Frank et al. 1988; Raskin 1974) because they are more willing to endure the side effects.

Men, on the other hand, are less willing to tolerate drug-induced sexual dysfunction, most certainly exacerbated by male stereotypes and perceptions of masculinity (Clarke et al. 2015). This ADR is not necessarily more common in either men or women, but it does appear to cause a more extensive amount of distress in men, who tend to report drug-associated sexual dysfunction and consider discontinuation of the implicated drug for this reason more often than women (Montejo et al. 2019). ADD-induced sexual dysfunction is predominantly associated with serotonergic ADDs, especially SSRIs and SSNRIs, and unfortunately may also persist even once the drug in question has been discontinued (Bala et al. 2018). Higher availability of serotonin stimulates postsynaptic 5-HT receptors, such as the subtype 2 A, which are though to significantly contribute to ADD-associated sexual dysfunction (Rothmore 2020). Mirtazapine, an antagonist at 5-HT_2A_ receptors, on the other hand, has a lower risk for sexual dysfunction (Montejo et al. 2001) and add-on treatment of mirtazapine has shown some benefit in the amelioration of sexual dysfunction induced by other ADDs (Michelson et al. 2002). Bupropion, which is void of serotonergic effects, appears to be associated with the lowest rate of ADD-associated sexual dysfunction (Clayton et al. 2014). While bupropion is favorable in patients hoping to avoid both weight gain and sexual dysfunction (Zimmerman et al. 2005), pharmacoepidemiologic studies have found a slightly higher utilization among men (Seifert et al. 2021).

Agomelatine, an ADD also thought to be generally well-tolerated apart from rare cases of hepatotoxicity, may show wider acceptance due to its lower rates of ADD-associated sexual dysfunction and weight gain (Plesničar 2014). In contrast to bupropion, agomelatine has been found to have a slightly higher utilization among women (Seifert et al. 2021).

### Effects of sex and gender on placebo- and nocebo-response to antidepressant therapy

All ADDs consistently outperform placebo in depressed patients compared to placebo, but with an average treatment effect of *d* = 0.3, their efficacy is limited (Cipriani et al. 2018) and raises concerns regarding clinical relevance (2 points on the 52-point Hamilton Rating Scale for Depression [HAMD-17]) (Hengartner and Plöderl 2018). Placebo effects substantially contribute to antidepressant response, accounting for up to 82% of treatment response (Kirsch et al. 2008), raising the question whether these effects are also modulated by sex and gender (Enck and Klosterhalfen 2019). A review of placebo effects in antidepressant RCTs was unable to report a clear difference between men and women regarding magnitude of response (Mora et al. 2011). A review of trials in patients with persistent depressive disorder found a higher rate of adverse effects in placebo arms (i. e., nocebo response) when the proportion of females in the trial was lower. However, this evidence is weakened by a series of confounding factors (Mora et al. 2011). Among other psychiatric disorders, higher placebo response rates in women have been reported for bipolar mania (Yildiz et al. 2011) and questionably in schizophrenia (Mallinckrodt et al. 2010).

There is some cross-domain evidence of gender- and sex-dependent placebo and nocebo effects in relation to analgesia (Vambheim and Flaten 2017), suggesting that males show a stronger placebo response to analgesia than women, especially when verbally induced (e. g., when the patient’s expectations are shaped by verbal suggestions regarding the drug’s superior efficacy). Females, on the other hand, appear to be more responsive to conditioning-based placebo response to analgesia, which occurs when a patient associates a certain treatment with symptom relief after previous experiences. Women also appear more prone to nocebo responses to analgesia, indicating that negative expectations lead to side effects (Vambheim and Flaten 2017).

### Effects of sex and gender differences in symptom presentation of depression on antidepressant response

Depression is a highly heterogeneous clinical syndrome presenting with changes in mood, appetite, sleep, energy levels, cognition and psychomotor activity. A diagnosis is made when at least five symptoms are present for a minimum of two weeks, represent a change from previous functioning and result in clinically relevant distress. The presentation of symptoms and their respective severity is unique to each affected person with potentially over 200 different symptom presentations (Drysdale et al. 2017; Farmer and McGuffin 1989; Zimmerman et al. 2015). Some research has been put forth in the effort to identify different “biotypes” of depression (Tozzi et al. 2024) and how these might respond preferentially to specific treatment options including pharmacotherapy and psychotherapy (Drysdale et al. 2017; Hack et al. 2023), although some authors suggest that this approach is not currently not convincing (Arnow et al. 2015; Dinga et al. 2019). Moreover, most patients with depression suffer from comorbid mental disorders, including personality disorders, anxiety disorders and substance use disorders (Steffen et al. 2020). These considerations give rise to yet another question: Does either sex/gender tend to present with a presentation of depression or comorbidities that are more (or less) responsive to ADDs? While the discussion of this matter is worthwhile, a comprehensive review would go beyond the scopes of the present narrative review, therefore, only a small and inevitably incomplete selection of aspects will be presented in the following.

Depression in women often features “typical” symptoms that are included in the diagnostic criteria according to the Diagnostic and Statistical Manual of Mental Disorders, 5th Edition (DSM-V) (American Psychiatric Association 2013) or the International Classification of Disease, 10th Edition (ICD-10)(World Health Organization 1992). These include low mood, changes in appetite and sleep disturbances (Cavanagh et al. 2017). While others symptoms of depression seem to fail to significantly respond to ADD treatment, low mood has shown the most consistent response to SSRIs (Hieronymus et al. 2016). These two observations may account for the superior efficacy of SSRIs in women in some studies (Kornstein et al. 2000; Khan et al. 2005; Berlanga and Flores-Ramos 2006; Young et al. 2009), though it remains speculative. Depressed women are also more likely to be diagnosed with comorbid anxiety disorders than men (Simonds and Whiffen 2003). Conveniently, several ADDs, especially from the group of SSRIs and SSNRIs, are also indicated in the treatment of anxiety disorders (Ströhle et al. 2018). This, however, does not necessarily successfully translate into clinical practice, as comorbid anxiety disorders, especially generalized anxiety disorders, are not only characterized by a higher symptom severity, but also a poorer response to treatment (Dold et al. 2017).

Depressed men, on the other hand, are more likely to present with a different symptom profile which comprises substance use as well as risk taking and poor impulse control (Cavanagh et al. 2017). Interestingly, though highly prevalent among men (Cavanagh et al. 2017), these symptoms are not listed among the diagnostic criteria of the DSM-V (American Psychiatric Association 2013) or ICD-10 (World Health Organization 1992), which is an entirely different issue in itself. When these symptoms are present, they may not be receptive to standard pharmacological treatment options, such as SSRIs, and require a more integrative treatment approach. In fact, efficacious pharmacological treatment options for substance use disorders are limited (Reed et al. 2015). Moreover, comorbid substance use tends to yield poorer treatment outcomes in general (Hunt et al. 2020) and is associated with lower adherence to treatment (Herbeck et al. 2005).

## Summary and clinical implications

Despite seemingly convincing plausibility that sex and gender modulate response to ADDs via sex hormone effects on serotonergic and noradrenergic neurotransmission and pharmacokinetics, the clinical meaningfulness of these findings remain modest. Even when these biologically based theoretical observations are actionable, the treatment recommendations are limited to a very narrowly defined patient group, such as estrogen replacement in conjunction with fluoxetine in perimenopausal women with depression or allopregnanolone in postpartum depression. Other factors that mediate ADD response and tolerability, including ADRs, remain essential when treating with ADDs and the ADRs men and women are prepared to tolerate seem to differ, especially weight gain and sexual dysfunction. As ADD response appears to significantly be modulated by placebo response, it is particularly unfortunate that research in this field is lacking in regards to the impact of sex and gender. This, of course, does not necessarily mean that biologically and gender-based differences in ADD response and tolerability do not exist, it does however prompt further research, especially RCTs. Future studies should systematically integrate sex as a key variable in order to better understand potential sex and gender differences and derive treatment implications from them.

## Data Availability

Not applicable.

## Abbreviations

5-HT: Serotonin
ADD: Antidepressant drug
ADR: Adverse drug reaction
AMPA: α-Amino-3-hydroxy-5-methyl-4-isoxazolepropionic acid
BDNF: Brain-derived neurotrophic factor
CNS: Central nervous system
CYP: Cytochrome P450
DAT: Dopamine transporter
DHEA: Dehydroepiandrosterone
DHEA-S: Dehydroepiandrosterone sulfate
DSM: Diagnostic and Statistical Manual of Mental Disorders, 5th Edition
FDA: United State Food and Drug Administration
GABA: Gamma-aminobutyric acid
HAMD-17: Hamilton Rating Scale for Depression
ICD-10: International Classification of Disease, 10th Edition
MAO: Monoamine oxidase
MAOI: Monoamine oxidase inhibitor
NaSSA: Noradrenergic and specific serotonergic antidepressant
NDRI: Norepinephrine-dopamine reuptake inhibitor
NET: Norepinephrine transporter
NE: Norepinephrine
NMDA: N-methyl-D-aspartate
NRI: Norepinephrine reuptake inhibitor
RCT: Randomized controlled trial
SARI: Serotonin antagonist and reuptake inhibitor
SERT: Serotonin transporter
SSNRI: Selective serotonin-norepinephrine reuptake inhibitor
SSRI: Selective serotonin reuptake inhibitor
TCA: Tricyclic antidepressant
TRT: Testosterone replacement therapy

## References

[CR1] Almeida OP, Yeap BB, Hankey GJ, Jamrozik K, Flicker L (2008) Low free testosterone concentration as a potentially treatable cause of depressive symptoms in older men. Arch Gen Psychiatry 65(3):283–289. 10.1001/archgenpsychiatry.2007.33 18316674 10.1001/archgenpsychiatry.2007.33

[CR2] Alonso-Pedrero L, Bes-Rastrollo M, Marti A (2019) Effects of antidepressant and antipsychotic use on weight gain: a systematic review. Obes Rev 20 (12):1680–1690. 10.1111/obr.12934 10.1111/obr.12934 31524318

[CR3] American Psychiatric Association (2013) Diagnostic and statistical manual of mental disorders, 5th edn. 10.1176/appi.books.9780890425596

[CR4] Arnow BA, Blasey C, Williams LM, Palmer DM, Rekshan W, Schatzberg AF, Etkin A, Kulkarni J, Luther JF, Rush AJ (2015) Depression subtypes in predicting antidepressant response: a report from the iSPOT-D trial. Am J Psychiatry 172(8):743–750. 10.1176/appi.ajp.2015.14020181 25815419 10.1176/appi.ajp.2015.14020181

[CR5] Bala A, Nguyen HMT, Hellstrom WJG (2018) Post-SSRI sexual dysfunction: a literature review. Sex Med Rev 6(1):29–34. 10.1016/j.sxmr.2017.07.002 28778697 10.1016/j.sxmr.2017.07.002

[CR6] Baldinger P, Kranz GS, Haeusler D, Savli M, Spies M, Philippe C, Hahn A, Höflich A, Wadsak W, Mitterhauser M, Lanzenberger R, Kasper S (2014) Regional differences in SERT occupancy after acute and prolonged SSRI intake investigated by brain PET. Neuroimage 88:252–262. 10.1016/j.neuroimage.2013.10.002 24121201 10.1016/j.neuroimage.2013.10.002

[CR7] Bendis PC, Zimmerman S, Onisiforou A, Zanos P, Georgiou P (2024) The impact of estradiol on serotonin, glutamate, and dopamine systems. Front Neurosci 18:1348551. 10.3389/fnins.2024.1348551 38586193 10.3389/fnins.2024.1348551 PMC10998471

[CR8] Berlanga C, Flores-Ramos M (2006) Different gender response to serotonergic and noradrenergic antidepressants. A comparative study of the efficacy of citalopram and reboxetine. J Affect Disord 95(1–3):119–123. 10.1016/j.jad.2006.04.029 16782204 10.1016/j.jad.2006.04.029

[CR9] Berry-Bibee EN, Kim MJ, Simmons KB, Tepper NK, Riley HE, Pagano HP, Curtis KM (2016) Drug interactions between hormonal contraceptives and psychotropic drugs: a systematic review. Contraception 94(6):650–667. 10.1016/j.contraception.2016.07.011 27444984 10.1016/j.contraception.2016.07.011 PMC11283812

[CR10] Bhasin S, Seidman S (2019) Testosterone treatment of depressive disorders in men: too much smoke, not enough high-quality evidence. JAMA Psychiatr 76(1):9–10. 10.1001/jamapsychiatry.2018.2661 10.1001/jamapsychiatry.2018.2661 30428087

[CR11] Billups SJ, Delate T, Dugan D (2009) Evaluation of risk factors for elevated tricyclic antidepressant plasma concentrations. Pharmacoepidemiol Drug Saf 18(3):253–257. 10.1002/pds.1697 19148878 10.1002/pds.1697

[CR12] Bommersbach TJ, Rosenheck RA, Petrakis IL, Rhee TG (2022) Why are women more likely to attempt suicide than men? Analysis of lifetime suicide attempts among US adults in a nationally representative sample. J Affect Disord 311:157–164. 10.1016/j.jad.2022.05.096 35598742 10.1016/j.jad.2022.05.096

[CR13] Brabete AC, Greaves L, Maximos M, Huber E, Li A, Lê M-L (2022) A sex- and gender-based analysis of adverse drug reactions: a scoping review of pharmacovigilance databases. Pharmaceuticals 15(3):298 35337096 10.3390/ph15030298 PMC8950058

[CR14] Bromberger JT, Epperson CN (2018) Depression during and after the perimenopause: impact of hormones, genetics, and environmental determinants of disease. Obstet Gynecol Clin North Am 45(4):663–678. 10.1016/j.ogc.2018.07.007 30401549 10.1016/j.ogc.2018.07.007 PMC6226029

[CR15] Cavanagh A, Wilson CJ, Kavanagh DJ, Caputi P (2017) Differences in the expression of symptoms in men versus women with depression: a systematic review and meta-analysis. Harv Rev Psychiatry 25(1):29–38. 10.1097/hrp.0000000000000128 28059934 10.1097/HRP.0000000000000128

[CR16] Celada P, Puig MV, Amargós-Bosch M, Adell A, Artigas F (2004) The therapeutic role of 5-HT_1A_ and 5-HT_2A_ receptors in depression. J Psychiatry Neurosci 29(4):252–265 15309042 PMC446220

[CR17] Christiansen DM, McCarthy MM, Seeman MV (2022) Where sex meets gender: how sex and gender come together to cause sex differences in mental illness. Front Psychiatry. 10.3389/fpsyt.2022.856436 35836659 10.3389/fpsyt.2022.856436 PMC9273892

[CR18] Cipolla-Neto J, Amaral FG, Soares J, Maria J, Gallo CC, Furtado A, Cavaco JE, Gonçalves I, Santos CRA, Quintela T (2022) The crosstalk between melatonin and sex steroid hormones. Neuroendocrinology 112(2):115–129. 10.1159/000516148 33774638 10.1159/000516148

[CR19] Cipriani A, Furukawa TA, Salanti G, Chaimani A, Atkinson LZ, Ogawa Y, Leucht S, Ruhe HG, Turner EH, Higgins JPT, Egger M, Takeshima N, Hayasaka Y, Imai H, Shinohara K, Tajika A, Ioannidis JPA, Geddes JR (2018) Comparative efficacy and acceptability of 21 antidepressant drugs for the acute treatment of adults with major depressive disorder: a systematic review and network meta-analysis. Lancet 391(10128):1357–1366. 10.1016/s0140-6736(17)32802-7 29477251 10.1016/S0140-6736(17)32802-7 PMC5889788

[CR20] Cirrincione LR, Huang KJ (2021) Sex and gender differences in clinical pharmacology: implications for transgender medicine. Clin Pharmacol Ther 110(4):897–908. 10.1002/cpt.2234 33763856 10.1002/cpt.2234 PMC8518665

[CR21] Clarke MJ, Marks ADG, Lykins AD (2015) Effect of normative masculinity on males’ dysfunctional sexual beliefs, sexual attitudes, and perceptions of sexual functioning. J Sex Res 52(3):327–337. 10.1080/00224499.2013.860072 24558985 10.1080/00224499.2013.860072

[CR22] Clayton AH, El Haddad S, Iluonakhamhe JP, Ponce Martinez C, Schuck AE (2014) Sexual dysfunction associated with major depressive disorder and antidepressant treatment. Expert Opin Drug Saf 13(10):1361–1374. 10.1517/14740338.2014.951324 25148932 10.1517/14740338.2014.951324

[CR23] Coquoz A, D. R, and Stute P (2022) Impact of progesterone on the gastrointestinal tract: a comprehensive literature review. Climacteric 25 (4):337-361. 10.1080/13697137.2022.2033203 10.1080/13697137.2022.2033203 35253565

[CR24] Dahl ML, Bertilsson L, Nordin C (1996) Steady-state plasma levels of nortriptyline and its 10-hydroxy metabolite: relationship to the CYP2D6 genotype. Psychopharmacol 123(4):315–319. 10.1007/BF02246640 10.1007/BF02246640 8867869

[CR25] Damoiseaux VA, Proost JH, Jiawan VC, Melgert BN (2014) Sex differences in the pharmacokinetics of antidepressants: influence of female sex hormones and oral contraceptives. Clin Pharmacokinet 53(6):509–519. 10.1007/s40262-014-0145-2 24859034 10.1007/s40262-014-0145-2

[CR26] Del Río JP, Alliende MI, Molina N, Serrano FG, Molina S, Vigil P (2018) Steroid hormones and their action in women’s brains: the importance of hormonal balance. Front Public Health 6:141. 10.3389/fpubh.2018.00141 29876339 10.3389/fpubh.2018.00141 PMC5974145

[CR27] Dinga R, Schmaal L, Penninx BWJH, van Tol MJ, Veltman DJ, van Velzen L, Mennes M, van der Wee NJA, Marquand AF (2019) Evaluating the evidence for biotypes of depression: Methodological replication and extension of Drysdale et al. (2017). NeuroImage: Clinical 22:101796. 10.1016/j.nicl.2019.101796 10.1016/j.nicl.2019.101796 PMC6543446 30935858

[CR28] Dold M, Bartova L, Souery D, Mendlewicz J, Serretti A, Porcelli S, Zohar J, Montgomery S, Kasper S (2017) Clinical characteristics and treatment outcomes of patients with major depressive disorder and comorbid anxiety disorders - results from a European multicenter study. J Psychiatr Res 91:1–13. 10.1016/j.jpsychires.2017.02.020 28284107 10.1016/j.jpsychires.2017.02.020

[CR29] Drysdale AT, Grosenick L, Downar J, Dunlop K, Mansouri F, Meng Y, Fetcho RN, Zebley B, Oathes DJ, Etkin A, Schatzberg AF, Sudheimer K, Keller J, Mayberg HS, Gunning FM, Alexopoulos GS, Fox MD, Pascual-Leone A, Voss HU, Casey BJ, Dubin MJ, Liston C (2017) Resting-state connectivity biomarkers define neurophysiological subtypes of depression. Nat Med 23(1):28–38. 10.1038/nm.4246 27918562 10.1038/nm.4246 PMC5624035

[CR30] Duman RS, Aghajanian GK, Sanacora G, Krystal JH (2016) Synaptic plasticity and depression: new insights from stress and rapid-acting antidepressants. Nat Med 22(3):238–249. 10.1038/nm.4050 26937618 10.1038/nm.4050 PMC5405628

[CR31] Eder J, Simon MS, Glocker C, Musil R (2023) Gewichtszunahme unter Therapie mit Psychopharmaka. Nervenarzt 94(9):859–869. 10.1007/s00115-023-01534-z 37672085 10.1007/s00115-023-01534-z

[CR32] Eker SS, Kirli S, Akkaya C, Cangur S, Sarandol A (2009) Are there differences between serotonergic, noradrenergic and dual acting antidepressants in the treatment of depressed women? World J Biol Psychiatry 10(4–2):400–408. 10.1080/15622970903131886 19670086 10.1080/15622970903131886

[CR33] Enck P, Klosterhalfen S (2019) Does sex/gender play a role in placebo and nocebo effects? Conflicting evidence from clinical trials and experimental studies. Front Neurosci. 10.3389/fnins.2019.00160 30886569 10.3389/fnins.2019.00160 PMC6409330

[CR34] Entsuah AR, Huang H, Thase ME (2001) Response and remission rates in different subpopulations with major depressive disorder administered venlafaxine, selective serotonin reuptake inhibitors, or placebo. J Clin Psychiatry 62(11):869–877. 10.4088/jcp.v62n1106 11775046 10.4088/jcp.v62n1106

[CR35] Fakhoury M (2016) Revisiting the serotonin hypothesis: implications for major depressive disorders. Mol Neurobiol 53(5):2778–2786. 10.1007/s12035-015-9152-z 25823514 10.1007/s12035-015-9152-z

[CR36] Farmer A, McGuffin P (1989) The classification of the depressions. Contemporary confusion revisited. Br J Psychiatry 155:437–443. 10.1192/bjp.155.4.437 2692762 10.1192/bjp.155.4.437

[CR37] Favril L, Yu R, Uyar A, Sharpe M, Fazel S (2022) Risk factors for suicide in adults: systematic review and meta-analysis of psychological autopsy studies. Evid Based Ment Health 25(4):148–155. 10.1136/ebmental-2022-300549 36162975 10.1136/ebmental-2022-300549 PMC9685708

[CR38] Frackiewicz EJ, Sramek JJ, Cutler NR (2000) Gender differences in depression and antidepressant pharmacokinetics and adverse events. Ann Pharmacother 34(1):80–88. 10.1345/aph.18465 10669189 10.1345/aph.18465

[CR39] Frank E, Carpenter LL, Kupfer DJ (1988) Sex differences in recurrent depression: are there any that are significant? Am J Psychiatry 145(1):41–45. 10.1176/ajp.145.1.41 3337291 10.1176/ajp.145.1.41

[CR40] Freeman EW (2015) Depression in the menopause transition: risks in the changing hormone milieu as observed in the general population. Womens Midlife Health 1(1):2. 10.1186/s40695-015-0002-y 30766689 10.1186/s40695-015-0002-y PMC6214217

[CR41] Gates ML, Wilkins T, Ferguson E, Walker V, Bradford RK, Yoo W (2016) Gender and race disparities in weight gain among offenders prescribed antidepressant and antipsychotic medications. Health Justice 4:6. 10.1186/s40352-016-0037-7 27340612 10.1186/s40352-016-0037-7 PMC4877425

[CR42] Gex-Fabry M, Balant-Gorgia AE, Balant LP, Garrone G (1990) Clomipramine metabolism Model-based analysis of variability factors from drug monitoring data. Clin Pharmacokinet 19(3):241–255. 10.2165/00003088-199019030-00007 2394063 10.2165/00003088-199019030-00007

[CR43] Gill H, Gill B, El-Halabi S, Chen-Li D, Lipsitz O, Rosenblat JD, Van Rheenen TE, Rodrigues NB, Mansur RB, Majeed A, Lui LMW, Nasri F, Lee Y, McIntyre RS (2020) Antidepressant medications and weight change: a narrative review. Obes 28(11):2064–2072. 10.1002/oby.22969 10.1002/oby.22969 33022115

[CR44] Glantz MD, Bharat C, Degenhardt L, Sampson NA, Scott KM, Lim CCW, Al-Hamzawi A, Alonso J, Andrade LH, Cardoso G, De Girolamo G, Gureje O, He Y, Hinkov H, Karam EG, Karam G, Kovess-Masfety V, Lasebikan V, Lee S, Levinson D, McGrath J, Medina-Mora M-E, Mihaescu-Pintia C, Mneimneh Z, Moskalewicz J, Navarro-Mateu F, Posada-Villa J, Rapsey C, Stagnaro JC, Tachimori H, Ten Have M, Tintle N, Torres Y, Williams DR, Ziv Y, Kessler RC (2020) The epidemiology of alcohol use disorders cross-nationally: findings from the world mental health surveys. Addict Behav 102:106128. 10.1016/j.addbeh.2019.106128 31865172 10.1016/j.addbeh.2019.106128 PMC7416527

[CR45] Global Burden of Disease (2021). https://vizhub.healthdata.org/gbd-results/. Accessed Oct 27 2024

[CR46] Haack S, Seeringer A, Thürmann PA, Becker T, Kirchheiner J (2009) Sex-specific differences in side effects of psychotropic drugs: genes or gender? Pharmacogenomics 10(9):1511–1526. 10.2217/pgs.09.102 19761372 10.2217/pgs.09.102

[CR47] Hack LM, Tozzi L, Zenteno S, Olmsted AM, Hilton R, Jubeir J, Korgaonkar MS, Schatzberg AF, Yesavage JA, O’Hara R, Williams LM (2023) A cognitive biotype of depression linking symptoms, behavior measures, neural circuits, and differential treatment outcomes: a prespecified secondary analysis of a randomized clinical trial. JAMA Netw Open 6(6):e2318411–e2318411. 10.1001/jamanetworkopen.2023.18411 37318808 10.1001/jamanetworkopen.2023.18411 PMC10273022

[CR48] Hajós M, Fleishaker JC, Filipiak-Reisner JK, Brown MT, Wong EHF (2004) The selective norepinephrine reuptake inhibitor antidepressant reboxetine: pharmacological and clinical profile. CNS Drug Rev 10(1):23–44. 10.1111/j.1527-3458.2004.tb00002.x 14978512 10.1111/j.1527-3458.2004.tb00002.x PMC6741733

[CR49] Hamilton J (1996) Sex and treatment of depression. Psychopharmacology and women: sex, gender, and hormones. American Psychiatric Association Press, pp 241–260

[CR50] Hansen CH, Larsen LW, Sørensen AM, Halling-Sørensen B, Styrishave B (2017) The six most widely used selective serotonin reuptake inhibitors decrease androgens and increase estrogens in the H295R cell line. Toxicol in Vitro 41:1–11. 10.1016/j.tiv.2017.02.001 28179152 10.1016/j.tiv.2017.02.001

[CR51] Hasnain M, Vieweg WVR (2013) Weight considerations in psychotropic drug prescribing and switching. Postgrad Med 125(5):117–129. 10.3810/pgm.2013.09.2706 24113670 10.3810/pgm.2013.09.2706

[CR52] Hauger RL, Saelzler UG, Pagadala MS, Panizzon MS (2022) The role of testosterone, the androgen receptor, and hypothalamic-pituitary-gonadal axis in depression in ageing men. Rev Endocr Metab Disord 23(6):1259–1273. 10.1007/s11154-022-09767-0 36418656 10.1007/s11154-022-09767-0 PMC9789012

[CR53] Hengartner MP, Plöderl M (2018) Statistically significant antidepressant-placebo differences on subjective symptom-rating scales do not prove that the drugs work: effect size and method bias matter! Front Psychiatry 9:517. 10.3389/fpsyt.2018.00517 30386270 10.3389/fpsyt.2018.00517 PMC6199395

[CR54] Herbeck DM, Fitek DJ, Svikis DS, Montoya ID, Marcus SC, West JC (2005) Treatment compliance in patients with comorbid psychiatric and substance use disorders. Am J Addict 14(3):195–207. 10.1080/10550490590949488 16019970 10.1080/10550490590949488 PMC2599916

[CR55] Hernández-Hernández OT, Martínez-Mota L, Herrera-Pérez JJ, Jiménez-Rubio G (2019) Role of estradiol in the expression of genes involved in serotonin neurotransmission: implications for female depression. Curr Neuropharmacol 17(5):459–471. 10.2174/1570159x16666180628165107 29956632 10.2174/1570159X16666180628165107 PMC6520586

[CR56] Hiemke C, Bergemann N, Clement HW, Conca A, Deckert J, Domschke K, Eckermann G, Egberts K, Gerlach M, Greiner C, Gründer G, Haen E, Havemann-Reinecke U, Hefner G, Helmer R, Janssen G, Jaquenoud E, Laux G, Messer T, Mössner R, Müller MJ, Paulzen M, Pfuhlmann B, Riederer P, Saria A, Schoppek B, Schoretsanitis G, Schwarz M, Gracia MS, Stegmann B, Steimer W, Stingl JC, Uhr M, Ulrich S, Unterecker S, Waschgler R, Zernig G, Zurek G, Baumann P (2018) Consensus guidelines for therapeutic drug monitoring in neuropsychopharmacology: update 2017. Pharmacopsychiatry 51(1–02):9–62. 10.1055/s-0043-116492 28910830 10.1055/s-0043-116492

[CR57] Hieronymus F, Emilsson JF, Nilsson S, Eriksson E (2016) Consistent superiority of selective serotonin reuptake inhibitors over placebo in reducing depressed mood in patients with major depression. Mol Psychiatry 21(4):523–530. 10.1038/mp.2015.53 25917369 10.1038/mp.2015.53 PMC4804177

[CR58] Hildebrandt MG, Steyerberg EW, Stage KB, Passchier J, Kragh-Soerensen P (2003) Are gender differences important for the clinical effects of antidepressants? Am J Psychiatry 160(9):1643–1650. 10.1176/appi.ajp.160.9.1643 12944340 10.1176/appi.ajp.160.9.1643

[CR59] Horstmann A, Busse FP, Mathar D, Müller K, Lepsien J, Schlögl H, Kabisch S, Kratzsch J, Neumann J, Stumvoll M, Villringer A, Pleger B (2011) Obesity-related differences between women and men in brain structure and goal-directed behavior. Front Hum Neurosci 5:58. 10.3389/fnhum.2011.00058 21713067 10.3389/fnhum.2011.00058 PMC3114193

[CR60] Hunt GE, Malhi GS, Lai HMX, Cleary M (2020) Prevalence of comorbid substance use in major depressive disorder in community and clinical settings, 1990–2019: systematic review and meta-analysis. J Affect Disord 266:288–304. 10.1016/j.jad.2020.01.141 32056890 10.1016/j.jad.2020.01.141

[CR61] Huybrechts KF, Palmsten K, Mogun H, Kowal M, Avorn J, Setoguchi-Iwata S, Hernández-Díaz S (2013) National trends in antidepressant medication treatment among publicly insured pregnant women. Gen Hosp Psychiatry 35(3):265–271. 10.1016/j.genhosppsych.2012.12.010 23374897 10.1016/j.genhosppsych.2012.12.010 PMC4077674

[CR62] Hyde JS, Mezulis AH (2020) Gender differences in depression: biological, affective, cognitive, and sociocultural factors. Harv Rev Psychiatry 28(1):4–13. 10.1097/hrp.0000000000000230 31913978 10.1097/HRP.0000000000000230

[CR63] Jeong H (2010) Altered drug metabolism during pregnancy: hormonal regulation of drug-metabolizing enzymes. Expert Opin Drug Metab Toxicol 6(6):689–699. 10.1517/17425251003677755 20367533 10.1517/17425251003677755 PMC3686288

[CR64] Johnson DP, Whisman MA (2013) Gender differences in rumination: a meta-analysis. Pers Individ Dif 55(4):367–374. 10.1016/j.paid.2013.03.019 24089583 10.1016/j.paid.2013.03.019 PMC3786159

[CR65] Kalayjian A, Laszlo K, Fassler M, Schonrock Z, Delarose KE, Ly AM, English CD (2003) Cirrincione LR (2024) Patterns of psychotropic medication prescribing and potential drug-hormone interactions among transgender and gender-diverse adults within 2 years of hormone therapy. J Am Pharm Assoc 64(1):283-289.e282. 10.1016/j.japh.2023.10.005 10.1016/j.japh.2023.10.005 PMC10873097 37839699

[CR66] Kashuba AD, Nafziger AN (1998) Physiological changes during the menstrual cycle and their effects on the pharmacokinetics and pharmacodynamics of drugs. Clin Pharmacokinet 34(3):203–218. 10.2165/00003088-199834030-00003 9533982 10.2165/00003088-199834030-00003

[CR67] Khan A, Brodhead AE, Schwartz KA, Kolts RL, Brown WA (2005) Sex differences in antidepressant response in recent antidepressant clinical trials. J Clin Psychopharmacol 25(4):318–324. 10.1097/01.jcp.0000168879.03169.ce 16012273 10.1097/01.jcp.0000168879.03169.ce

[CR68] Kirsch I, Deacon BJ, Huedo-Medina TB, Scoboria A, Moore TJ, Johnson BT (2008) Initial severity and antidepressant benefits: a meta-analysis of data submitted to the food and drug administration. PLoS Med 5(2):e45. 10.1371/journal.pmed.0050045 18303940 10.1371/journal.pmed.0050045 PMC2253608

[CR69] Koceva A, Herman R, Janez A, Rakusa M, Jensterle M (2024) Sex- and gender-related differences in obesity: from pathophysiological mechanisms to clinical implications. Int J Mol Sci. 10.3390/ijms25137342 39000449 10.3390/ijms25137342 PMC11242171

[CR70] Kokras N, Dalla C, Papadopoulou-Daifoti Z (2011) Sex differences in pharmacokinetics of antidepressants. Expert Opin Drug Metab Toxicol 7(2):213–226. 10.1517/17425255.2011.544250 21192772 10.1517/17425255.2011.544250

[CR71] König H, König HH, Konnopka A (2019) The excess costs of depression: a systematic review and meta-analysis. Epidemiol Psychiatr Sci 29:e30. 10.1017/s2045796019000180 30947759 10.1017/S2045796019000180 PMC8061284

[CR72] Kornstein SG, Schatzberg AF, Thase ME, Yonkers KA, McCullough JP, Keitner GI, Gelenberg AJ, Davis SM, Harrison WM, Keller MB (2000) Gender differences in treatment response to sertraline versus imipramine in chronic depression. Am J Psychiatry 157(9):1445–1452. 10.1176/appi.ajp.157.9.1445 10964861 10.1176/appi.ajp.157.9.1445

[CR73] Kornstein SG, Clayton AH, Soares CN, Padmanabhan SK, Guico-Pabia CJ (2010) Analysis by age and sex of efficacy data from placebo-controlled trials of desvenlafaxine in outpatients with major depressive disorder. J Clin Psychopharmacol 30(3):294–299. 10.1097/JCP.0b013e3181dcb594 20473066 10.1097/JCP.0b013e3181dcb594

[CR74] Kornstein SG, Pedersen RD, Holland PJ, Nemeroff CB, Rothschild AJ, Thase ME, Trivedi MH, Ninan PT, Keller MB (2014) Influence of sex and menopausal status on response, remission, and recurrence in patients with recurrent major depressive disorder treated with venlafaxine extended release or fluoxetine: analysis of data from the PREVENT study. J Clin Psychiatry 75(1):62–68. 10.4088/JCP.12m07841 24345717 10.4088/JCP.12m07841

[CR75] Kuehner C (2003) Gender differences in unipolar depression: an update of epidemiological findings and possible explanations. Acta Psychiatr Scand 108(3):163–174. 10.1034/j.1600-0447.2003.00204.x 12890270 10.1034/j.1600-0447.2003.00204.x

[CR76] Kuehner C (2017) Why is depression more common among women than among men? Lancet Psychiatry 4(2):146–158. 10.1016/s2215-0366(16)30263-2 27856392 10.1016/S2215-0366(16)30263-2

[CR77] Lacroix C, Maurier A, Largeau B, Destere A, Thillard E-M, Drici M, Micallef J, Jonville-Bera AP (2023) Sex differences in adverse drug reactions: are women more impacted? Therapies 78(2):175–188. 10.1016/j.therap.2022.10.002 10.1016/j.therap.2022.10.002 36283857

[CR78] Lanzenberger R, Kranz GS, Haeusler D, Akimova E, Savli M, Hahn A, Mitterhauser M, Spindelegger C, Philippe C, Fink M, Wadsak W, Karanikas G, Kasper S (2012) Prediction of SSRI treatment response in major depression based on serotonin transporter interplay between median raphe nucleus and projection areas. Neuroimage 63(2):874–881. 10.1016/j.neuroimage.2012.07.023 22828162 10.1016/j.neuroimage.2012.07.023

[CR79] LeGates TA, Kvarta MD, Thompson SM (2019) Sex differences in antidepressant efficacy. Neuropsychopharmacology 44(1):140–154. 10.1038/s41386-018-0156-z 30082889 10.1038/s41386-018-0156-z PMC6235879

[CR80] Li S, Zhang X, Cai Y, Zheng L, Pang H, Lou L (2023) Sex difference in incidence of major depressive disorder: an analysis from the Global Burden of Disease Study 2019. Ann Gen Psychiatry 22(1):53. 10.1186/s12991-023-00486-7 38087353 10.1186/s12991-023-00486-7 PMC10714584

[CR81] Lim GY, Tam WW, Lu Y, Ho CS, Zhang MW, Ho RC (2018) Prevalence of depression in the community from 30 countries between 1994 and 2014. Sci Rep 8(1):2861. 10.1038/s41598-018-21243-x 29434331 10.1038/s41598-018-21243-x PMC5809481

[CR82] Liu Q, He H, Yang J, Feng X, Zhao F, Lyu J (2020) Changes in the global burden of depression from 1990 to 2017: findings from the global burden of disease study. J Psychiatr Res. 10.1016/j.jpsychires.2019.08.002 31439359 10.1016/j.jpsychires.2019.08.002

[CR83] Maguire JL, Mennerick S (2024) Neurosteroids: mechanistic considerations and clinical prospects. Neuropsychopharmacology 49(1):73–82. 10.1038/s41386-023-01626-z 37369775 10.1038/s41386-023-01626-z PMC10700537

[CR84] Mallinckrodt CH, Zhang L, Prucka WR, Millen BA (2010) Signal detection and placebo response in schizophrenia: parallels with depression. Psychopharmacol Bull 43(1):53–72 20581800

[CR85] Martinowich K, Lu B (2008) Interaction between BDNF and serotonin: role in mood disorders. Neuropsychopharmacol 33(1):73–83. 10.1038/sj.npp.1301571 10.1038/sj.npp.1301571 17882234

[CR86] McEwen BS, Milner TA (2017) Understanding the broad influence of sex hormones and sex differences in the brain. J Neurosci Res 95(1–2):24–39. 10.1002/jnr.23809 27870427 10.1002/jnr.23809 PMC5120618

[CR87] Medeiros GC, Demo I, Goes FS, Zarate CA, Gould TD (2024) Personalized use of ketamine and esketamine for treatment-resistant depression. Transl Psychiatry 14(1):481. 10.1038/s41398-024-03180-8 39613748 10.1038/s41398-024-03180-8 PMC11607365

[CR88] Michelson D, Kociban K, Tamura R, Morrison MF (2002) Mirtazapine, yohimbine or olanzapine augmentation therapy for serotonin reuptake-associated female sexual dysfunction: a randomized, placebo controlled trial. J Psychiatr Res 36(3):147–152. 10.1016/s0022-3956(01)00060-7 11886692 10.1016/s0022-3956(01)00060-7

[CR89] Molero P, Ramos-Quiroga JA, Martin-Santos R, Calvo-Sánchez E, Gutiérrez-Rojas L, Meana JJ (2018) Antidepressant efficacy and tolerability of ketamine and esketamine: a critical review. CNS Drugs 32(5):411–420. 10.1007/s40263-018-0519-3 29736744 10.1007/s40263-018-0519-3

[CR90] Möller-Leimkühler AM, Bottlender R, Strauss A, Rutz W (2004) Is there evidence for a male depressive syndrome in inpatients with major depression? J Affect Disord 80(1):87–93. 10.1016/s0165-0327(03)00051-x 15094262 10.1016/S0165-0327(03)00051-X

[CR91] Montejo AL, Calama J, Rico-Villademoros F, Montejo L, González-García N, Pérez J (2019) A real-world study on antidepressant-associated sexual dysfunction in 2144 outpatients: the SALSEX I study. Arch Sex Behav 48(3):923–933. 10.1007/s10508-018-1365-6 30790204 10.1007/s10508-018-1365-6

[CR92] Montejo AL, Llorca G, Izquierdo JA, Rico-Villademoros F (2001) Incidence of sexual dysfunction associated with antidepressant agents: a prospective multicenter study of 1022 outpatients. Spanish Working Group for the Study of Psychotropic-Related Sexual Dysfunction. J Clin Psychiatry 62 Suppl 3:10–21 11229449

[CR93] Mora MS, Nestoriuc Y, Rief W (2011) Lessons learned from placebo groups in antidepressant trials. Philos Trans R Soc Lond B Biol Sci 366(1572):1879–1888. 10.1098/rstb.2010.0394 21576145 10.1098/rstb.2010.0394 PMC3130402

[CR94] Mu E, Kulkarni J (2022) Hormonal contraception and mood disorders. Aust Prescr 45(3):75–79. 10.18773/austprescr.2022.025 35755988 10.18773/austprescr.2022.025 PMC9218393

[CR95] Mwinyi J, Cavaco I, Pedersen RS, Persson A, Burkhardt S, Mkrtchian S, Ingelman-Sundberg M (2010) Regulation of CYP2C19 expression by estrogen receptor α: implications for estrogen-dependent inhibition of drug metabolism. Mol Pharmacol 78(5):886–894. 10.1124/mol.110.065540 20675569 10.1124/mol.110.065540

[CR96] Nashwan AJ, Rehan ST, Imran L, Abbas SG, Khan SF (2024) Exploring the clinical potentials of zuranolone in managing postpartum depression: a new therapeutic horizon. Prog Neuropsychopharmacol Biol Psychiatry 132:110983. 10.1016/j.pnpbp.2024.110983 38412941 10.1016/j.pnpbp.2024.110983

[CR97] Nautiyal KM, Hen R (2017) Serotonin receptors in depression: from A to B. F1000Res 6:123. 10.12688/f1000research.9736.1 28232871 10.12688/f1000research.9736.1 PMC5302148

[CR98] Nenezic N, Kostic S, Strac DS, Grunauer M, Nenezic D, Radosavljevic M, Jancic J, Samardzic J (2023) Dehydroepiandrosterone (DHEA): pharmacological effects and potential therapeutic application. Mini Rev Med Chem 23(8):941–952. 10.2174/1389557522666220919125817 36121077 10.2174/1389557522666220919125817

[CR99] Parker G, Parker K, Austin MP, Mitchell P, Brotchie H (2003) Gender differences in response to differing antidepressant drug classes: two negative studies. Psychol Med 33(8):1473–1477. 10.1017/s0033291703007918 14672256 10.1017/s0033291703007918

[CR100] Patwardhan V, Gil GF, Arrieta A, Cagney J, DeGraw E, Herbert ME, Khalil M, Mullany EC, O’Connell EM, Spencer CN, Stein C, Valikhanova A, Gakidou E, Flor LS (2024) Differences across the lifespan between females and males in the top 20 causes of disease burden globally: a systematic analysis of the Global Burden of Disease Study 2021. Lancet Public Health 9(5):e282–e294. 10.1016/S2468-2667(24)00053-7 38702093 10.1016/S2468-2667(24)00053-7 PMC11080072

[CR101] Paulzen M, Schoretsanitis G (2023) Psychopharmacotherapy during pregnancy and breastfeeding-Part I: focus on pregnancy : support options by using therapeutic drug monitoring. Nervenarzt 94(9):786–798. 10.1007/s00115-023-01528-x 37460797 10.1007/s00115-023-01528-x

[CR102] Payne JL, Maguire J (2019) Pathophysiological mechanisms implicated in postpartum depression. Front Neuroendocrinol 52:165–180. 10.1016/j.yfrne.2018.12.001 30552910 10.1016/j.yfrne.2018.12.001 PMC6370514

[CR103] Peixoto C, Grande AJ, Mallmann MB, Nardi AE, Cardoso A, Veras AB (2018) Dehydroepiandrosterone (DHEA) for depression: a systematic review and meta-analysis. CNS Neurol Disord Drug Targets 17(9):706–711. 10.2174/1871527317666180817153914 30124161 10.2174/1871527317666180817153914

[CR104] Plesničar BK (2014) Efficacy and tolerability of agomelatine in the treatment of depression. Patient Prefer Adherence 8:603–612. 10.2147/ppa.S42789 24833894 10.2147/PPA.S42789 PMC4014359

[CR105] Pletzer B, Comasco E, Hidalgo-Lopez E, Lacreuse A, Derntl B (2023) Editorial: Effects of hormonal contraceptives on the brain. Front Endocrinol. 10.3389/fendo.2023.1129203 10.3389/fendo.2023.1129203 PMC9927394 36798667

[CR106] Quitkin FM, Stewart JW, McGrath PJ, Taylor BP, Tisminetzky MS, Petkova E, Chen Y, Ma G, Klein DF (2002) Are there differences between women’s and men’s antidepressant responses? Am J Psychiatry 159(11):1848–1854. 10.1176/appi.ajp.159.11.1848 12411218 10.1176/appi.ajp.159.11.1848

[CR107] Racagni G, Riva MA, Molteni R, Musazzi L, Calabrese F, Popoli M, Tardito D (2011) Mode of action of agomelatine: synergy between melatonergic and 5-HT2C receptors. World J Biol Psychiatry 12(8):574–587. 10.3109/15622975.2011.595823 21999473 10.3109/15622975.2011.595823

[CR108] Raskin A (1974) Age-sex differences in response to antidepressant drugs. J Nerv Ment Dis 159(2):120–130. 10.1097/00005053-197408000-00006 4850465 10.1097/00005053-197408000-00006

[CR109] Reddy DS, Mbilinyi RH, Estes E (2023) Preclinical and clinical pharmacology of brexanolone (allopregnanolone) for postpartum depression: a landmark journey from concept to clinic in neurosteroid replacement therapy. Psychopharmacol 240(9):1841–1863. 10.1007/s00213-023-06427-2 10.1007/s00213-023-06427-2 PMC10471722 37566239

[CR110] Reed K, Edward D, Jenny K, Strang J (2015) Pharmacological treatments for drug misuse and dependence. Expert Opin Pharmacother 16(3):325–333. 10.1517/14656566.2015.983472 25413001 10.1517/14656566.2015.983472

[CR111] Regenass W, Möller M, Harvey BH (2018) Studies into the anxiolytic actions of agomelatine in social isolation reared rats: role of corticosterone and sex. J Psychopharmacol 32(2):134–145. 10.1177/0269881117735769 29082818 10.1177/0269881117735769

[CR112] Rothmore J (2020) Antidepressant-induced sexual dysfunction. Med J Aust 212(7):329–334. 10.5694/mja2.50522 32172535 10.5694/mja2.50522

[CR113] Rybaczyk LA, Bashaw MJ, Pathak DR, Moody SM, Gilders RM, Holzschu DL (2005) An overlooked connection: serotonergic mediation of estrogen-related physiology and pathology. BMC Womens Health 5:12. 10.1186/1472-6874-5-12 16368009 10.1186/1472-6874-5-12 PMC1327664

[CR114] Schneider M, Pauwels P, Toto S, Bleich S, Grohmann R, Heinze M, Greiner T (2020) Severe weight gain as an adverse drug reaction of psychotropics: data from the AMSP project between 2001 and 2016. Eur Neuropsychopharmacol 36:60–71. 10.1016/j.euroneuro.2020.05.001 32536570 10.1016/j.euroneuro.2020.05.001

[CR115] Schoretsanitis G, Spigset O, Stingl JC, Deligiannidis KM, Paulzen M, Westin AA (2020) The impact of pregnancy on the pharmacokinetics of antidepressants: a systematic critical review and meta-analysis. Expert Opin Drug Metab Toxicol 16(5):431–440. 10.1080/17425255.2020.1750598 32238008 10.1080/17425255.2020.1750598 PMC7323120

[CR116] Schoretsanitis G, M. DK, Michael P, Edoardo S, and de Leon J (2022) Drug-drug interactions between psychotropic medications and oral contraceptives. Expert Opin Drug Metab Toxicol 18 (6):395-411 10.1080/17425255.2022.2106214 10.1080/17425255.2022.2106214 35876180

[CR117] Schumacher M, Mattern C, Ghoumari A, Oudinet JP, Liere P, Labombarda F, Sitruk-Ware R, De Nicola AF, Guennoun R (2014) Revisiting the roles of progesterone and allopregnanolone in the nervous system: resurgence of the progesterone receptors. Prog Neurobiol 113:6–39. 10.1016/j.pneurobio.2013.09.004 24172649 10.1016/j.pneurobio.2013.09.004

[CR118] Seeman MV (2020) Men and women respond differently to antipsychotic drugs. Neuropharmacol 163:107631. 10.1016/j.neuropharm.2019.05.008 10.1016/j.neuropharm.2019.05.008 31077728

[CR119] Seifert J, Führmann F, Reinhard MA, Engel RR, Bernegger X, Bleich S, Stübner S, Rüther E, Toto S, Grohmann R, Sieberer M, Greil W (2021) Sex differences in pharmacological treatment of major depressive disorder: results from the AMSP pharmacovigilance program from 2001 to 2017. J Neural Transm (Vienna) 128(6):827–843. 10.1007/s00702-021-02349-5 33977402 10.1007/s00702-021-02349-5 PMC8205885

[CR120] Semahegn A, Torpey K, Manu A, Assefa N, Tesfaye G, Ankomah A (2020) Psychotropic medication non-adherence and its associated factors among patients with major psychiatric disorders: a systematic review and meta-analysis. Sys Rev 9(1):17. 10.1186/s13643-020-1274-3 10.1186/s13643-020-1274-3 PMC6966860 31948489

[CR121] Shi P, Yang A, Zhao Q, Chen Z, Ren X, Dai Q (2021) A hypothesis of gender differences in self-reporting symptom of depression: implications to solve under-diagnosis and under-treatment of depression in males. Front Psychiatry 12:589687. 10.3389/fpsyt.2021.589687 34759845 10.3389/fpsyt.2021.589687 PMC8572815

[CR122] Shorey S, Chee CYI, Ng ED, Chan YH, Tam WWS, Chong YS (2018) Prevalence and incidence of postpartum depression among healthy mothers: a systematic review and meta-analysis. J Psychiatr Res 104:235–248. 10.1016/j.jpsychires.2018.08.001 30114665 10.1016/j.jpsychires.2018.08.001

[CR123] Simonds VM, Whiffen VE (2003) Are gender differences in depression explained by gender differences in co-morbid anxiety? J Affect Disord77 (3):197–202. 10.1016/S0165-0327(02)00113-1 10.1016/s0165-0327(02)00113-1 14612219

[CR124] Souza-Teodoro LH, Andrade LHSG, Carvalho LA (2022) Could dehydroepiandrosterone (DHEA) be a novel target for depression? J Affect Disord Rep 8:100340. 10.1016/j.jadr.2022.100340

[CR125] Souza-Teodoro LH, Davies NM, Warren HR, Andrade LHSG, Carvalho LA (2024) DHEA and response to antidepressant treatment: a Mendelian randomization analysis. J Psychiatr Res 173:151–156. 10.1016/j.jpsychires.2024.02.049 38531145 10.1016/j.jpsychires.2024.02.049

[CR126] Sripada RK, Welsh RC, Marx CE, Liberzon I (2014) The neurosteroids allopregnanolone and dehydroepiandrosterone modulate resting-state amygdala connectivity. Hum Brain Mapp 35(7):3249–3261. 10.1002/hbm.22399 24302681 10.1002/hbm.22399 PMC4739102

[CR127] Stahl SM (2009) Mechanism of action of trazodone: a multifunctional drug. CNS Spectr 14(10):536–546. 10.1017/S1092852900024020 20095366 10.1017/s1092852900024020

[CR128] Stahl SM (2021) Stahl’s essential psychopharmacology: neuroscientific basis and practical applications. Cambridge University Press

[CR129] Steffen A, Nübel J, Jacobi F, Bätzing J, Holstiege J (2020) Mental and somatic comorbidity of depression: a comprehensive cross-sectional analysis of 202 diagnosis groups using German nationwide ambulatory claims data. BMC Psychiatry 20(1):142. 10.1186/s12888-020-02546-8 32228541 10.1186/s12888-020-02546-8 PMC7106695

[CR130] Ströhle A, Gensichen J, Domschke K (2018) The diagnosis and treatment of anxiety disorders. Dtsch Arztebl Int 155(37):611–620. 10.3238/arztebl.2018.0611 30282583 10.3238/arztebl.2018.0611 PMC6206399

[CR131] Sundell KA, Gissler M, Petzold M, Waern M (2011) Antidepressant utilization patterns and mortality in Swedish men and women aged 20–34 years. Eur J Clin Pharmacol 67(2):169–178. 10.1007/s00228-010-0933-z 21063694 10.1007/s00228-010-0933-z

[CR132] Thiels C, Linden M, Grieger F, Leonard J (2005) Gender differences in routine treatment of depressed outpatients with the selective serotonin reuptake inhibitor sertraline. Int Clin Psychopharmacol 20(1):1–7. 10.1097/00004850-200501000-00001 15602108 10.1097/00004850-200501000-00001

[CR133] Tozzi L, Zhang X, Pines A, Olmsted AM, Zhai ES, Anene ET, Chesnut M, Holt-Gosselin B, Chang S, Stetz PC, Ramirez CA, Hack LM, Korgaonkar MS, Wintermark M, Gotlib IH, Ma J, Williams LM (2024) Personalized brain circuit scores identify clinically distinct biotypes in depression and anxiety. Nat Med 30(7):2076–2087. 10.1038/s41591-024-03057-9 38886626 10.1038/s41591-024-03057-9 PMC11271415

[CR134] Tseng PT, Chiu HJ, Suen MW, Zeng BS, Wu MK, Tu YK, Hung KC, Wu YC, Su KP, Li DJ, Chen TY, Stubbs B, Carvalho AF, Solmi M, Thompson T, Caruso MG, Matsuoka YJ, Chen YW, Lin PY, Sun CK, Cheng YS, Shiue YL (2023) Pharmacological interventions and hormonal therapies for depressive symptoms in peri- and post-menopausal women: a network meta-analysis of randomized controlled trials. Psychiatry Res 326:115316. 10.1016/j.psychres.2023.115316 37399764 10.1016/j.psychres.2023.115316

[CR135] Twenge JM, Nolen-Hoeksema S (2002) Age, gender, race, socioeconomic status, and birth cohort differences on the children's depression inventory: a meta-analysis. J Abnorm Psychol 111 (4):578-58810.1037/0021-843x.111.4.578 10.1037//0021-843x.111.4.578 12428771

[CR136] Ullsperger JM, Nikolas MA (2017) A meta-analytic review of the association between pubertal timing and psychopathology in adolescence: are there sex differences in risk? Psychol Bull 143(9):903–938. 10.1037/bul0000106 28530427 10.1037/bul0000106

[CR137] Unterecker S, Hiemke C, Greiner C, Haen E, Jabs B, Deckert J, Pfuhlmann B (2012) The effect of age, sex, smoking and co-medication on serum levels of venlafaxine and O-desmethylvenlafaxine under naturalistic conditions. Pharmacopsychiatry 45(6):229–235. 10.1055/s-0031-1301366 22426847 10.1055/s-0031-1301366

[CR138] Unterecker S, Riederer P, Proft F, Maloney J, Deckert J, Pfuhlmann B (2013) Effects of gender and age on serum concentrations of antidepressants under naturalistic conditions. J Neural Transm 120(8):1237–1246. 10.1007/s00702-012-0952-2 23254926 10.1007/s00702-012-0952-2

[CR139] Vambheim SM, Flaten MA (2017) A systematic review of sex differences in the placebo and the nocebo effect. J Pain Res 10:1831–1839. 10.2147/jpr.S134745 28831271 10.2147/JPR.S134745 PMC5548268

[CR140] Vartolomei MD, Kimura S, Vartolomei L, Shariat SF (2020) Systematic review of the impact of testosterone replacement therapy on depression in patients with late-onset testosterone deficiency. Eur Urol Focus 6(1):170–177. 10.1016/j.euf.2018.07.006 30017901 10.1016/j.euf.2018.07.006

[CR141] Want SC (2009) Meta-analytic moderators of experimental exposure to media portrayals of women on female appearance satisfaction: social comparisons as automatic processes. Body Image 6(4):257–269. 10.1016/j.bodyim.2009.07.008 19716779 10.1016/j.bodyim.2009.07.008

[CR142] Winkler D, Pjrek E, Kasper S (2005) Gender-specific symptoms of depression and anger attacks. J Mens Health Gend 3(1):19–24. 10.1016/j.jmhg.2005.05.004

[CR143] Woolley CS, McEwen BS (1993) Roles of estradiol and progesterone in regulation of hippocampal dendritic spine density during the estrous cycle in the rat. J Comp Neurol 336(2):293–306. 10.1002/cne.903360210 8245220 10.1002/cne.903360210

[CR144] World Health Organization (1992) The ICD-10 classification of mental and behavioural disorders: diagnostic criteria for research. World Health Organization, Geneva

[CR145] Yildiz A, Vieta E, Tohen M, Baldessarini RJ (2011) Factors modifying drug and placebo responses in randomized trials for bipolar mania. Int J Neuropsychopharmacol 14(7):863–875. 10.1017/s1461145710001641 21299919 10.1017/S1461145710001641

[CR146] Young EA, Kornstein SG, Marcus SM, Harvey AT, Warden D, Wisniewski SR, Balasubramani GK, Fava M, Trivedi MH, John Rush A (2009) Sex differences in response to citalopram: a STAR*D report. J Psychiatr Res 43(5):503–511. 10.1016/j.jpsychires.2008.07.002 18752809 10.1016/j.jpsychires.2008.07.002 PMC2681489

[CR147] Zhang H, Cui M, Cao J-L, Han M-H (2022) The role of beta-adrenergic receptors in depression and resilience. Biomedicines 10(10):2378 36289638 10.3390/biomedicines10102378 PMC9598882

[CR148] Zheng Y, Chen X, Benet LZ (2016) Reliability of in vitro and in vivo methods for predicting the effect of P-glycoprotein on the delivery of antidepressants to the brain. Clin Pharmacokinet 55(2):143–167. 10.1007/s40262-015-0310-2 26293617 10.1007/s40262-015-0310-2 PMC5674979

[CR149] Zimmerman M, Posternak MA, Attiullah N, Friedman M, Boland RJ, Baymiller S, Berlowitz SL, Rahman S, Uy KK, Singer S, Chelminski I (2005) Why isn’t bupropion the most frequently prescribed antidepressant? J Clin Psychiatry 66(5):603–610. 10.4088/jcp.v66n0510 15889947 10.4088/jcp.v66n0510

[CR150] Zimmerman M, Ellison W, Young D, Chelminski I, Dalrymple K (2015) How many different ways do patients meet the diagnostic criteria for major depressive disorder? Compr Psychiatry 56:29–34. 10.1016/j.comppsych.2014.09.007 25266848 10.1016/j.comppsych.2014.09.007

